# Association between circulating leukocytes and arrhythmias: Mendelian randomization analysis in immuno-cardiac electrophysiology

**DOI:** 10.3389/fimmu.2023.1041591

**Published:** 2023-04-05

**Authors:** Yuxiao Chen, Lian Lou, Xuan Zhang, Luyang Jin, Yao Chen, Lele Chen, Zhihang Li, Fen Zhang, Ting Fu, Shenjiang Hu, Jian Yang

**Affiliations:** ^1^ Department of Cardiology, The First Affiliated Hospital, Zhejiang University School of Medicine, Hangzhou, China; ^2^ Department of Gynecology, The First Affiliated Hospital, Zhejiang University School of Medicine, Hangzhou, China; ^3^ Department of Cardiology, Jinhua People’s Hospital, Jinhua, China; ^4^ Department of Cardiology, Yiwu Central Hospital, Jinhua, China

**Keywords:** leukocyte, lymphocyte, arrhythmia, atrioventricular block, Mendelian randomization

## Abstract

**Background:**

Cardiac arrhythmia is a common disease associated with high mortality and morbidity. Circulating leukocyte counts, which serve as a biomarker for assessing systemic immune status, have been linked to arrhythmias in observational studies. However, observational studies are plagued by confounding factors and reverse causality, whether alterations in circulating leukocyte components are causally associated with arrhythmias remains uncertain. The present study explored this question based on genetic evidence.

**Methods and findings:**

We performed Mendelian randomization (MR) analysis to evaluate whether alterations in leukocyte counts affect aggregated risk of all types of arrhythmia or risk of five specific types of arrhythmia. Single-nucleotide polymorphisms serving as proxies for leukocyte differential counts were retrieved from the Blood Cell Consortium, and statistical data on arrhythmias were obtained from the UK Biobank), FinnGenand a meta-analysis of genome-wide association studies for atrial fibrillation. We applied inverse variance-weighted method as the primary analysis, complemented by a series of sensitivity analyses. Bidirectional analyses were conducted to assess reverse causality. Finally, multivariable MR was performed to study the joint effects of multiple risk factors. We found that genetically predicted differential leukocyte counts were not significantly associated with aggregated occurrence of all types of arrhythmia. In contrast, each 1-standard deviation increase in lymphocyte count was associated with 46% higher risk of atrioventricular block (OR 1.46, 95% CI 1.11–1.93, p=0.0065). A similar effect size was observed across all MR sensitivity analyses, with no evidence of horizontal pleiotropy. Reverse MR analysis suggested that atrioventricular block was unlikely to cause changes in lymphocyte count. Primary MR analysis based on the inverse-variance weighted method suggested that changes in neutrophil count alter risk of right bundle branch block, and changes in basophil count alter risk of atrial fibrillation. However, these causal relationships were not robust in sensitivity analyses. We found no compelling evidence that neutrophil or lymphocyte counts cause atrial fibrillation.

**Conclusion:**

Our data support higher lymphocyte count as a causal risk factor for atrioventricular block. These results highlight the importance of immune cells in the pathogenesis of specific cardiac conduction disorders.

## 1 Introduction

Cardiac arrhythmia, a condition in which the heartbeat is irregular, too fast or too slow, is a relatively common heart disease. Some arrhythmias are brief and asymptomatic, while others are persistent and can lead to hemodynamic instability, thromboembolic events, and even cardiac sudden death, imposing a significant burden on healthcare systems. However, evidence on how to effectively prevent and treat arrhythmias, thus far, has been limited.

Although how exactly leukocytes participate in arrhythmogenesis is not fully understood, it is generally accepted that leukocytes might contribute to arrhythmia either directly through coupling to cardiomyocytes, or indirectly by producing cytokines and antibodies (1). Neutrophils, which are rarely found in the healthy myocardium, are rapidly recruited to the heart in response to stress signals, and they exert the arrhythmogenic effect by releasing myeloperoxidase (2) and lipocalin (3), promoting oxidative stress and interstitial fibrosis. Monocytes/macrophages are the most numerous leukocytes in the heart, and can effectively clear dysfunctional mitochondria, apoptotic cells and debris, thus preventing ventricular tachycardia and fibrillation after myocardial infarction (3). However, macrophages can also secrete cytokines like IL-1β, which then prolong the action potential duration of cardiomyocytes and induces arrhythmias (4). Recent research has uncovered a non-canonical leukocyte function for macrophages in cardiac electrical conduction, demonstrating that they can directly couple to conducting cardiomyocytes *via* gap junctions containing Cx43, altering their electrical properties (5). T lymphocytes elicit cell-mediated immunity, and subsets of T lymphocytes may produce cytokines like IFN-γ, IL-2 or IL-17, exacerbating the neutrophilic inflammation, then promote micro-scar formation among myocardial tissue, leading to insulating fibrosis (6). B lymphocytes can promote cardiac arrhythmias by means of autoantibodies targeting specific calcium, potassium, or sodium channels on the surface of cardiomyocytes (7). Last, less is known about the function of basophils and eosinophils in arrhythmias, but recent experimental data highlight that basophil-derived IL-14 plays an essential role in the heart, by balancing macrophage polarization. While eosinophils may play an anti-inflammatory and cardioprotective role after myocardial infarction, reducing cardiomyocyte death and inflammatory cell accumulation (8, 9). Due to these pioneering works, researchers have attempted to integrate electrophysiology and immunology, and a new terminology “immuno-cardiac electrophysiology” was introduced to highlight the emerging essential role of immune cells in arrhythmias (1, 10, 11).

Circulating leukocytes are crude markers of the systemic immunological status of individuals, and they can modulate local inflammatory responses. Cellular numbers are the most critical parameter for homeostasis of circulating immune cells. So far, some cross-sectional clinical surveys have linked circulating leukocyte counts to incidence of cardiac arrhythmias. In the CALIBER study of 775,231 individuals, high neutrophil count (12), low eosinophil count, and low lymphocyte count (13) were associated with ventricular arrhythmia. In the Framingham Heart Study, white blood cell counts correlated with risk of atrial fibrillation (14). Other studies have linked risk of atrial fibrillation to eosinophil count (15) and proportion of monocyte subsets (16). However, that literature does not definitively establish a role for leukocyte counts in the pathogenesis of arrhythmias because observational studies are prone to residual unmeasured confounding and reverse causation. Of particular concern is the potential for reverse causation. Atrial fibrillation itself might promote systemic inflammation during atrial remodeling and induce a spurious inverse association. In addition, observational studies have come to conflicting conclusions about the association of leukocyte counts with supraventricular tachycardia (17, 18). Therefore, evidence from observational studies alone is insufficient. The causal effect of leukocyte counts on the risk of arrhythmias remains unknown. And additional studies are needed to characterize the role of each immune cell subtype in different types of arrhythmias. Addressing these causal questions can accelerate the discovery of mechanisms underlying disease and open new prevention and treatment avenues.

Mendelian randomization (MR) is an epidemiologic approach that strives to address some key limitations of observational studies, such as confounding and reverse causation (19). It uses genetic variants, usually single-nucleotide polymorphisms (SNPs), as proxies for clinical interventions (as a result of exposure) in order to assess whether the genetic variants are associated with the outcome. In this way, MR supports inferences about causality (20), placing it at the interface between traditional observational epidemiology and interventional trials (21). MR should be robust to confounders, given that alleles are randomly distributed at conception, and it should be robust to reverse causation, since an individual’s genetic code is fixed at birth, before the outcome of interest. In the present study, two-sample MR was used to estimate whether leukocyte counts cause changes in arrhythmia risk, based on summary data in genome-wide association studies (GWAS).

## 2 Methods

### 2.1 Study design

For the current study, we conducted two-sample MR analysis of circulating leukocyte counts on arrhythmias using data from publicly available GWAS. The five subtypes of leukocytes were considered: neutrophils, eosinophils, basophils, monocytes, and lymphocytes. Arrythmia was defined as all types in aggregate or as one of the following five specific types: atrial fibrillation, atrioventricular block, left bundle branch block (LBBB), right bundle branch block (RBBB), and paroxysmal tachycardia.

All study procedures were performed in accordance with the World Medical Association Declaration of Helsinki ethical principles for medical research. Ethics approval was considered unnecessary for the present study because the included GWAS reported appropriate ethical approval from their respective institutions, and the present analyses were performed only on summary-level data.

### 2.2 Selection of genetic instruments for circulating leukocyte counts

We extracted summary statistics from the largest meta-analyzed GWAS data provided by the Blood Cell Consortium (22). The Blood Cell Consortium Phase 2 includes 563,946 European participants from 26 GWAS cohorts, after excluding patients with blood cancer, acute medical/surgical illness, myelodysplastic syndrome, bone marrow transplant, congenital/hereditary anemia, HIV, end-stage kidney disease, splenectomy, cirrhosis or extreme blood cell counts. An overview of the data sources is provided in **Supplementary Table S1**, and more detail is available in the original article (23).

SNPs associated with the counts of the five leukocyte counts were selected at the genome-wide significance level (*P*<5×10^–8^) and defined as genetic instruments. To ensure that SNPs were independent, a clumping procedure was performed, and the SNPs were pruned at a stringent linkage disequilibrium (LD) of *R* ^2^ < 0.001 within a 10,000-kb window.

The proportions of variance in respective leukocyte counts explained by the selected SNPs were estimated (**Supplementary Table S2**), and F-statistics were calculated as measures of instrument strength (24). The F value for all genetic instruments was > 10, ensuring that weak bias would be <10% at least 95% of the time (**Supplementary Table S2**).

### 2.3 Data sources for arrhythmia

To more thoroughly evaluate the association of leukocyte counts and the risk of arrhythmias, we aimed to include all eligible GWAS of arrhythmias by extensively searching the public Integrative Epidemiology Unit (IEU) GWAS database (https://gwas.mrcieu.ac.uk/). We selected GWAS with the largest samples, leading to seven GWAS whose summary statistics for different types of arrhythmias were used in the present study.

Genetic association estimates for the outcome of all types of arrhythmia were obtained from the UK Biobank (UK Biobank field ID 20002, value 1077), based on the UKB GWAS pipeline set up for the MRC IEU. We restricted the analytical cohort to individuals of European descent. Individuals with cardiac arrhythmia were identified *via* self-report during a face-to-face interview with a trained nurse. The GWAS dataset on atrial fibrillation was obtained from a meta-analysis comprising 1,030,836 participants of European ancestry (25). Cases of atrial fibrillation were defined as those patients with paroxysmal atrial fibrillation, permanent atrial fibrillation, or atrial flutter. Summary data for the other four types of arrhythmias (atrioventricular block, LBBB, RBBB, and paroxysmal tachycardia) were retrieved from the FinnGen project (release 2), where cases were defined as those assigned the corresponding ICD-10 diagnosis codes. Specifically, cases of atrioventricular block were defined as patients with first degree (ICD10:I440), second degree (I441), third degree atrioventricular block (I442) or other unspecified atrioventricular block (I443). LBBB included left anterior fascicular block (I444), left posterior fascicular block(I445), other fascicular block (I446) and unspecified LBBB (I447). While RBBB inlcuded right fascicular block (I450) and other RBBB (I451). And the term paroxysmal tachycardia referred to re-entry ventricular, supraventricular, ventricular tachycardia and unspecified paroxysmal tachycardia (I47). The FinnGen project included 102,739 Finnish participants and combined genetic data from Finnish biobanks and health records from Finnish health registries. Further details on data sources are included in **Supplementary Table S1**.

Prior to the MR analyses, we harmonized the SNPs identified from exposure GWAS with SNPs in outcome GWAS in order to align alleles on the same strand.

### 2.4 Statistical analyses

We used the inverse-variance weighted (IVW) method as the primary analysis. Then we applied a range of sensitivity analyses to assess the robustness of the IVW findings against potential violations, including MR-Egger, weighted median, MR-PRESSO and multivariable MR (MVMR) analyses. Although these methods have relatively low statistical efficiency on their own, they have different theoretical properties to control for different types of biases, and they are robust to certain assumption violations.

The IVW method (random effects model) can provide the greatest statistical power (26), assuming all genetic instruments are valid. This method is equivalent to a weighted linear regression of the SNP-exposure effects against the SNP-outcome effects, with the intercept constrained to zero. Owing to this constraint, it can lead to a relatively high rate of false positives in the presence of horizontal pleiotropy. Cochran’s Q statistic (27) from IVW analysis was used for global heterogeneity testing. Based on the notion that pleiotropy is one of the main sources of heterogeneity, low heterogeneity (Cochran’s Q p > 0.05) implies the minor possibility of pleiotropy.

MR-Egger regression is performed similarly as IVW, except the intercept is not fixed to zero (28). Therefore, the slope coefficient of MR-Egger regression gives an adjusted causal estimate, even when pleiotropy is present. The intercept of MR-Egger regression is an indicator of average pleiotropic effect across the genetic variants. An intercept of zero associated with *P* > 0.05 was considered evidence for absence of pleiotropic bias.

The weighted median method (29) is a consensus approach that takes the median of the ratio estimate distribution as the overall causal estimate. It has the advantage that it provides unbiased estimates when more than 50% of the weight comes from valid variants. It is less affected when a few genetic variants have pleiotropic effects, and it can be viewed as an implicit outlier removal approach.

The MR-PRESSO (30), a newly proposed MR method, is a variation on the IVW method. MR-PRESSO global test is used to assess the presence of overall horizontal pleiotropy. If pleiotropy is detected, the MR-PRESSO outlier test allows the detection of individual pleiotropic outliers through calculation of the residual sum of squares. Finally, the causal estimate is obtained by applying the IVW method to the genetic variants remaining after exclusion of outliers.

Steiger filtering (31), which computes the amount of variance each SNP explains in the exposure and in the outcome variable, identifies variant instruments that are likely to reflect reverse causation.

When significant horizontal pleiotropy was detected, we also used Cook’s distance to identify outliers. Cook’s distance identifies SNPs that exert disproportionate influences on the overall estimates as outliers.

MVMR, an extension of the standard MR approach, considers multiple correlated exposures within a single model, allowing the disentanglement of independent associations of each exposure with the outcome. This method was performed while considering associations of SNPs with diabetes mellitus (DM), hypertension, and coronary artery disease (CAD) as covariates in order to estimate the direct effects of leukocyte counts independently of risk factors known to influence risk of arrhythmia. Given the strong correlations between leukocyte subtypes, we also performed MVMR to determine the effect of each of the five leukocyte subtypes separately on arrhythmia, after adjusting for the effects of the other four subtypes.

We performed reverse-direction MR analysis to evaluate whether there is genetic evidence for the possibility that arrhythmia alters circulating leukocyte counts. Because we detected few genome-wide significant SNPs for arrhythmias (defined as p < 5×10^–8^), we used a less stringent statistical threshold (p<1×10^–5^) to select genetic instruments (32). In fact, we were unable to detect eligible SNPs associated with the aggregated occurrence of all types of arrhythmia, even at the suggestive level of p < 1×10^–5^, so this outcome was not included in the analysis. In this reverse-direction analysis, IVW, MR-Egger and weighted median analyses were performed as described above.

All statistical analyses were conducted using the TwoSampleMR, MendelianRandomization, and MR-PRESSO packages in R (version 4.0.3). Effect estimates for dichotomous outcomes were reported as odds ratios (ORs) with corresponding 95% confidence intervals (CIs).

### 2.5 Interpretation of results

Normally, Bonferroni-corrected p values are used to adjust for multiple testing. However, given the large number of arrhythmia outcomes and leukocyte counts in the study, we judged this correction procedure to be unnecessarily conservative (33). Therefore, we applied the conventional p value threshold of 0.05, and we interpreted p values near 0.05 with caution.

We considered casual associations to be strongly supported if the following four criteria were satisfied. (1) Primary IVW analysis gave a statistically significant causal estimate (p < 0.05). (2) All sensitivity analyses yielded concordant estimates, despite making different assumptions. (3) No evidence of unbalanced horizontal pleiotropy was observed, defined as p >0.05 for Cochran’s Q statistic, MR–Egger intercept test and MR-PRESSO global pleiotropy test. (4) No evidence of reverse causation from arrhythmias to leukocyte differential counts was observed, defined as p > 0.05 in the IVW, MR-Egger, and weighted median analyses in reverse MR analysis.

## 3 Results

### 3.1 Circulating leukocyte counts and heart arrhythmias: Primary results

**Figure 1:**
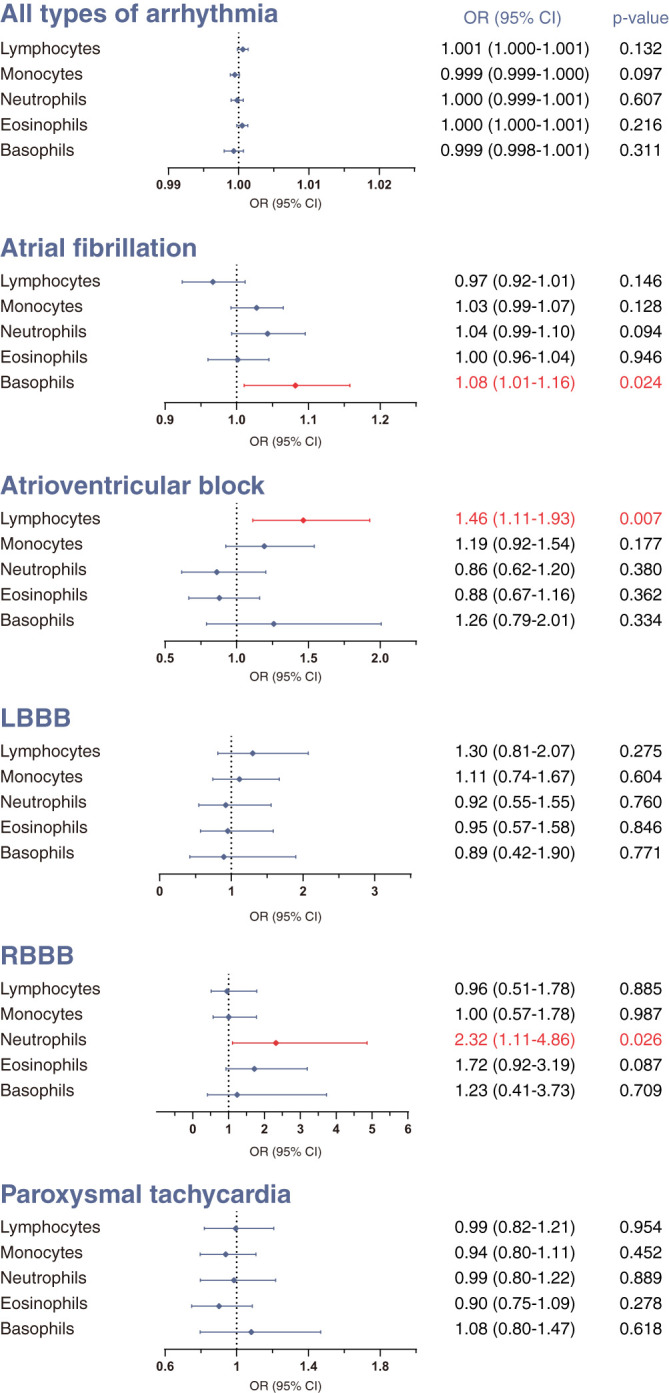
Mendelian randomization (MR) estimates derived from the inverse-variance weighted (IVW) method to assess the causal effect of genetically predicted differential leukocyte counts on arrhythmias. Statistical significance was defined as p<0.05. LBBB, left bundle-branch block; RBBB, right bundle-branch block; OR, odds ratio; CI, confidence interval.

First, we investigated the causal effect of each leukocyte subtype count on arrhythmias using IVW methods with multiplicative random effects. The IVW approach is recommended as the primary method in MR analysis because it is optimally efficient when all genetic variants are valid (34). The results of IVW analysis are presented in **Figure 1**.

We did not find clear evidence supporting causal effects of any leukocyte subtype counts on the overall occurrence of all-type arrhythmia (**Figure 1**). Nevertheless, there was evidence that different leukocyte subtype counts causally affected three specific types of arrhythmias. A genetically estimated 1-standard deviation increase in lymphocyte count was associated with 46% higher risk of atrioventricular block (OR 1.46, 95% CI 1.11–1.93, p=0.0065). We also found moderate evidence for causal effects of basophil count on atrial fibrillation (OR 1.08, 95% CI 1.01–1.58, p=0.0237), and neutrophil count on RBBB (OR 2.32, 95% CI 1.11–4.86, p=0.0259). No significant associations were observed for the other outcomes.

### 3.2 Sensitivity analyses of positive results

We assessed the robustness of the significant causal estimates from the above IVW analysis using sensitivity analyses. These sensitivity analyses are generally considered less powerful than the conventional IVW approach, but robust to different forms of biases (see Methods). Therefore we conducted MR-Egger, MR-PRESSO, weighted median, Steiger filtering and multivariable MR analyses on the following three combinations of exposure and outcome: (1) lymphocyte count and atrioventricular block, (2) neutrophil count and RBBB, and (3) basophil count and atrial fibrillation.

#### 3.2.1 Lymphocyte count and atrioventricular block

**Figure 2:**
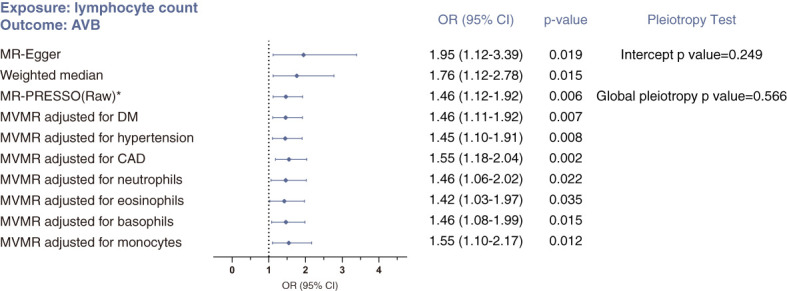
Sensitivity analyses of the causal association between lymphocyte count and risk of atrioventricular block using MR-Egger, weighted median, MR-PRESSO and multivariable MR (MVMR) analyses. *No outlier was detected. Statistical significance was defined as p<0.05.

Sensitivity analyses supported the causal link between lymphocyte count and atrioventricular block (**Figure 2**): the MR-Egger approach indicated an OR 1.95 (95% CI 1.12-3.39; p=0.019), and the weighted median approach indicated an OR 1.76 (95% CI 1.20-2.78; p=0.015). With respect to pleiotropy detection, Cochran’s Q test gave a p value of 0.586, suggesting no evidence of heterogeneity between genetic instruments and therefore no pleiotropy. Similarly, bias due to pleiotropy was not detectable in the IVW analyses, based on a p value of 0.249 for the MR-Egger intercept test and p value of 0.566 for MR-PRESSO global pleiotropy test. Additionally, the absence of outliers detected through the Steiger filtering reinforced this conclusion (**Supplementary Table S3**).

Using MVMR analysis, we confirmed this causal relationship after adjusting for risk factors of arrhythmia (CAD, DM and hypertension) and for effects from the other four subtypes of leukocytes (**Figure 2**).

#### 3.2.2 Neutrophil count and RBBB

**Figure 3:**
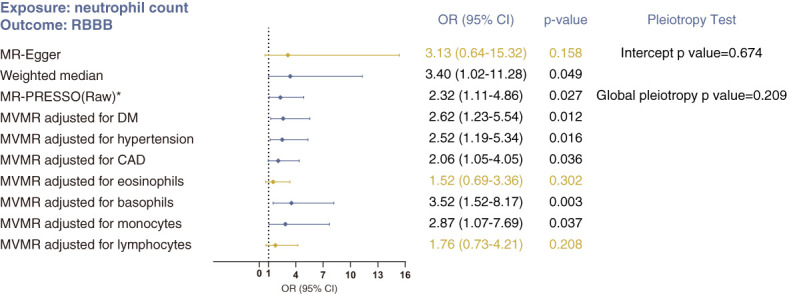
Sensitivity analyses of the causal association between neutrophil count and risk of RBBB using MR-Egger, weighted median, MR-PRESSO and MVMR analyses. *No outlier was detected. Statistical significance was defined as p<0.05.

The weighted median method (OR 3.40, 95% CI 1.02–11.28; p=0.049) and MR-Egger method (OR 3.13, 95% CI 0.64–15.32; p=0.158) produced results similar with those of the primary IVW analysis (**Figure 3**). But these CIs were wide and the p values near 0.05 or above 0.05, likely due to lack of statistical power. There was no indication of heterogeneity or pleiotropy in the corresponding Cochran’s Q test (p =0.200), MR-Egger intercept test (p=0. 674) or MR-PRESSO global pleiotropy test (p=0.209). And Steiger filtering did not detect any outliers (**Supplementary Table S3**). In MVMR analysis, accounting for counts of lymphocytes and eosinophils abolished the direct effect of neutrophil count on RBBB (**Figure 2**). Together, these analyses suggest no direct, independent effect of neutrophil counts on the risk of RBBB.

#### 3.2.3 Basophil count and atrial fibrillation

**Figure 4:**
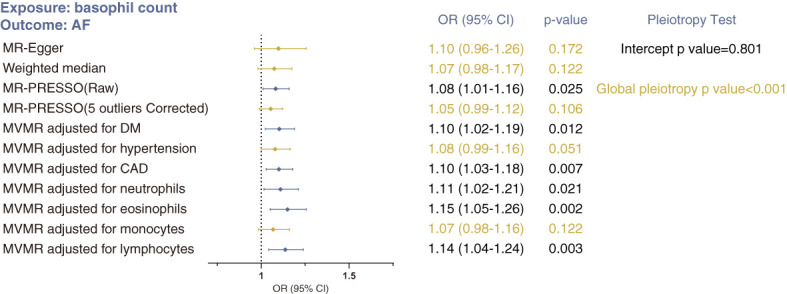
Sensitivity MR analyses of the causal association between basophil count and risk of atrial fibrillation using MR-Egger, weighted median, MR-PRESSO and MVMR analyses. Statistical significance was defined as p<0.05.

For basophil count and atrial fibrillation, the issue of horizontal pleiotropy is a particular concern (**Figure 4**). Although the intercept estimated from the MR-Egger regression was centered around zero (−0.0004, p=0.801), and Steiger filtering did not identify any outliers (**Supplementary Table S3**), we determined the presence of overall horizontal pleiotropy among all genetic instruments using MR-PRESSO (global pleiotropy p<0.001). After removing five outlier SNPs, the causal estimate of basophil count on atrial fibrillation no longer achieved statistical significance (MR-PRESSO outlier correction p=0.106). Similarly, effect estimates from MR-Egger and weighted median analyses were not significant. In conclusion, these analyses suggest that the estimate from IVW analysis may be strongly affected by pleiotropy, and that no compelling evidence exists in support of a causal association between basophil count and atrial fibrillation.

### 3.3 Sensitivity analyses of negative results

To reduce the incidence of false negative findings, sensitivity analyses (MR-Egger, weighted median, Steiger filtering, MR-PRESSO) were also performed to assess the validity of negative results. Empirically, we focused on the causal relationships of lymphocyte count or neutrophil count with atrial fibrillation.

For lymphocyte count and atrial fibrillation, both MR-Egger and MR-PRESSO methods gave negative, non-significant estimates similar to those of the IVW analysis (**Table 1**). Only weighted median analysis showed a significant, albeit small, effect. Steiger filtering identified one outlier SNP, but the results of above analysis remained essentially unchanged after removing the outlier (**Supplementary Table S3**). The finding from weighted median analysis alone is insufficient evidence. Overall, we conclude the absence of strong evidence for a causal association between lymphocyte count and risk of atrial fibrillation.

**Table 1:** Sensitivity MR analyses evaluating the causal effects of neutrophil and lymphocyte counts on atrial fibrillation.

Exposure	Outcome	Method	OR (95% CI)	p value	Pleiotropy test p value
Lymphocyte count	Atrial fibrillation	MR-Egger	0.99 (0.90-1.08)	0.762	0.66
		Weighted median	0.93 (0.88-0.99)	0.014	
		MR-PRESSO (Raw)	0.97 (0.92-1.01)	0.147	<0.001
		MR-PRESSO (remove 9 outliers)	0.97 (0.93-1.01)	0.129	
		IVW (remove 25 outliers identified by cook’s distance)	0.96 (0.92-1.00)	0.051	
Neutrophil count	Atrial fibrillation	MR-Egger	1.17 (1.05-1.29)	0.003	0.013
		Weighted median	1.09 (1.03-1.16)	0.006	
		MR-PRESSO (Raw)	1.04 (0.99-1.10)	0.095	<0.001
		MR-PRESSO (remove 9 outliers)	1.04 (0.99-1.09)	0.131	
		IVW (remove 19 outliers identified by cook’s distance)	1.04 (0.99-1.09)	0.17	

For neutrophil count and atrial fibrillation, the MR-Egger and weighted median analyses showed a statistically significant causal estimate, which was inconsistent with the IVW analysis (**Table 1**). These two methods are perceived as methods that have natural robustness to pleiotropy. Meanwhile, we found evidence of pleiotropy based on the p values for the MR-Egger intercept test (p=0.013) and MR-PRESSO global pleiotropy test(p<0.001), as well as evidence of substantial heterogeneity based on the p value for Cochran’s Q statistic (p<0.001). We suspect that pleiotropy biased the effect estimate towards null in the IVW analysis, even if pleiotropy more often biases estimates away from null. To remove potential pleiotropy as much as possible, we applied two additional different methods(MR-PRESSO outlier test and Cook’s distance) to further determine and exclude potential outliers. Using MR-PRESSO and Cook’s distance, we identified 9 and 19 outliers, respectively. After removing the outlier SNPs, the causal estimates still did not reach statistical significance (**Table 1**). In fact, the estimates were even smaller than before. Taken together, our analyses indicate no compelling evidence for a causal effect of neutrophil count on atrial fibrillation.

Sensitivity analyses of the other 29 exposure-outcome combinations yielded negative findings similar to those of the IVW analyses (**Supplementary Table S4**).

### 3.4 Reverse MR analysis to assess the effect of arrhythmias on leukocyte counts

To examine the possibility that reverse causation could be driving our findings, we performed extensive reverse MR analysis in which the risk of arrhythmia was the exposure and counts of the five leukocyte subtypes were the outcome. Although the IVW analysis showed that atrial fibrillation, paroxysmal tachycardia, LBBB and RBBB all had effects on the differential leukocyte counts, the effect sizes were so small that their practical significance is highly questionable (**Supplementary Table S5**). Moreover, these causal effects did not achieve statistical significance in either MR-Egger or weighted median analysis (**Supplementary Table S5**). Therefore, we did not found any robust evidence of reverse associations. In particular, we did not observe causal effects of atrioventricular block on lymphocyte count in IVW method (OR 1.001, 95% CI 0.998–1.004; *P*p=0.44) (**Supplementary Table S5**). Similar results were observed in MR-Egger and weighted median analyses (**Supplementary Table S5**).

## 4 Discussion

In this study, using large publicly available genomic datasets, we conducted MR analyses to investigate the causal effects of leukocyte counts on different types of arrhythmias. Our principal findings are that genetically determined high lymphocyte count increases risk of atrioventricular block. In contrast, we did not detect a significant causal effect of either neutrophil or lymphocyte count on risk of atrial fibrillation. Although sparse observational studies have reported relationships between leukocyte counts and some types of arrhythmias, the unique contribution of the present study is that we precisely investigated the association of each differential leukocyte count with five specific types of arrhythmias. In addition, we used MR methods, which help to minimize bias due to confounding factors and reverse causation, allowing us to draw conclusions about causal relationships, not merely associations.

Diversity is an intrinsic characteristic of the immune system, which exerts an important influence on an individual’s risk of developing immune mediated diseases. Although the abundance of circulating immune cells is particularly prone to change in the context of infection or injury, it has been demonstrated to be highly variable even among “healthy” individuals (35). Moreover, evidence has suggested that immune cell composition is associated with risks of cancer (36) and cardiovascular disease (12) among healthy people without prior corresponding diseases, although the exact causal relationship between immune cell composition changes and disease remains unclear. The analyses in the present study were carried out on data in the Blood Cell Consortium, for which mean leukocyte counts were within the normal range (22). Thus, our results may support the potential of leukocyte counts for predicting assessing arrhythmia risk in disease-free individuals.

High-degree atrioventricular block is the leading reason for pacemaker implantation. First-degree atrioventricular block, previously thought to be associated with a favorable prognosis, may actually be linked to adverse cardiovascular outcomes and increased mortality (37). However, due to the unknown mechanism of atrioventricular block, prevention and non-invasive treatment strategies are largely lacking in clinical practice. In particular, whether changes in circulating leukocyte components affect the risk of developing atrioventricular block remains unclear, as is the question of which types of leukocyte exert greater influence on atrioventricular block. Macrophages have been implicated in the disorder: they are abundant at the atrioventricular node and affect its physiological function though electrical coupling with cardiomyocytes (5). However, the current study did not find evidence supporting a causal effect of circulating monocyte count on atrioventricular block. We assume that this discrepancy stems from the fact that most cardiac macrophages, especially those resident in the atrioventricular node, populate the heart during embryogenesis and self-maintain locally with minimal exchange with the population of circulating monocytes (5, 38). On the other hand, our results revealed that genetically determined high lymphocyte count increases the risk of atrioventricular block. To the best of our knowledge, data on the impact of lymphocyte on atrioventricular block are scarce. The etiology of atrioventricular block is related to fibrosis of the conduction system (39), electrical remodeling of atrioventricular node myocytes (40, 41), and elevated vagal tone (42). Depending on the types of cells involved, it is speculated that lymphocytes may affect atrioventricular conduction in various ways. For instance, by secreting cytokines, lymphocytes can regulate monocyte/macrophage recruitment and differentiation (43). As previously mentioned, macrophages can directly affect the action potential of cardiomyocytes through gap junctions (5). Additionally, lymphocytes can promote fibroblast activation by secreting inflammatory mediators (44), leading to fibrosis in the atrioventricular node area and subsequent electrical isolation. Moreover, it may be possible that during cardiac injury, endogenous antigens in the conduction system are exposed, triggering the proliferation of autoreactive T and B cells and subsequent damage to atrioventricular node myocytes. Finally, it is worth investigating whether lymphocytes can directly couple to cardiomyocytes or produce autoantibodies that cross-react with ion channels in cardiomyocytes and ultimately affect their action potential. In conclusion, our results justify detailed studies into the role of lymphocytes in the pathogenesis of atrioventricular block, as well as their utility as a biomarker in disease risk assessment. Atrial fibrillation is the most common arrhythmia, and it increases the risk of stroke, heart failure and mortality (45). Previous observational studies have reported links between the disorder and high ratios of circulating neutrophils to lymphocytes (46, 47). Animal studies further support that atrial fibrillation involves atrial infiltration by neutrophils (48). However, we did not find any significant association between genetically predicted neutrophil or lymphocyte counts and atrial fibrillation. In particular, although our effect estimates for neutrophil counts were directionally concordant with the results from observational studies, the effect sizes were small and the CIs wide. These findings, coupled with inconsistent estimates from our various sensitivity analyses, lead us to conclude that genetically determined neutrophil counts do not substantially influence risk of atrial fibrillation. One potential reason for the differences between our work and previous epidemiological studies is that our MR analysis evaluated how *lifelong* exposure to increased leukocyte counts affected risk of atrial fibrillation (21). In contrast, observational studies typically have limited follow-up and may focus on short-term effects of leukocyte counts on the risk of postoperative atrial fibrillation (49). This study has limitations worth considering. First, it was restricted to a population of European descent for the sake of genetic homogeneity, so its generalizability to other ethnic groups is unclear. Second, lymphocytes are a diverse population of cells that have distinct phenotypic and functional properties. The aggregated count of all lymphocytes is far from fully representing the heterogeneous changes of lymphocyte subpopulations. Future studies should examine specific subsets of circulating lymphocytes, such as through fluorescence-activated cell sorting. Third, we were unable to distinguish different subtype of each kind of arrhythmias in our analysis, due to the lack of detailed original GWAS data. Fourth, no MR analysis can entirely exclude the influence of pleiotropic effects. Nevertheless, the observed consistency of effect estimates across multiple sensitivity analyses implies minimal confounding and bias. Fifth, this study did not encompass ventricular tachycardia or ventricular fibrillation, as large-scale population-based GWAS summary statistics on ventricular arrhythmias are currently unavailable. Recruiting patients with ventricular fibrillation in the setting of acute myocardial infarction is challenging when compared to the ease of recruitment of atrial fibrillation patients (50). Existing GWAS primarily focus on electrophysiological parameters that highly correlated with ventricular tachyarrhythmia, such as PR interval (51), QT interval (50, 52), or specific diseases like Brugada syndrome (53) or long QT syndrome (54), which are predominantly characterized by ventricular arrhythmias. Sixth, the “all types of arrhythmias” analyzed in the study represent a collection of phenotypes. It may introduce composition bias. This is because the proportion of each arrhythmia in the dataset is unknown, and changes in the proportion can significantly impact the causal effects, thereby reducing reproducibility. Furthermore, if the causal effects of leukocytes are opposite on different types of arrhythmias, they may mutually cancel out, resulting in inaccurate findings.

## 5 Conclusion

In conclusion, our study provides strong evidence of a causal effect of genetically high lymphocyte count on the risk of atrioventricular block. We failed to find evidence supporting a causal effect of lymphocyte or neutrophil count on atrial fibrillation. Our results provide insights into the role of systemic immune changes in the pathogenesis of arrhythmias.

## Data availability statement

The original contributions presented in the study are included in the article/**Supplementary Material**. Further inquiries can be directed to the corresponding authors.

## Ethics statement

Ethical review and approval was not required for the study on human participants in accordance with the local legislation and institutional requirements. Written informed consent for participation was not required for this study in accordance with the national legislation and the institutional requirements.

## Author contributions

YuC, JY and SH contributed to conception and design of the study. YuC, LJ, XZ, YaC, LC, FZ, ZL and TF performed the statistical analysis. YuC and LL wrote the first draft of the manuscript. YuC, LL, XZ and YaC wrote sections of the manuscript All authors contributed to the article and approved the submitted version.
