# Supplementary material for: Association between circulating leukocytes and arrhythmias: Mendelian randomization analysis in immuno-cardiac electrophysiology
Source: Front Immunol. 2023 Apr 5;14:1041591. doi: 10.3389/fimmu.2023.1041591 (PMC10113438; doi:10.3389/fimmu.2023.1041591)
Supplement: Supplementary file 1 [file DataSheet_1.pdf]

Table S1. Characteristics of the cohorts included in the MR study.

Phenotype	Description	Consortium/Study	Unit	Sample size
**Exposure data**				
Lymphocyte count	Aggregate count of lymphoid cells per unit volume of blood	Blood Cell Consortium	per nL	563,946
Monocyte count	Count of monocytes per unit volume of blood	Blood Cell Consortium	per nL	563,946
Neutrophil count	Count of neutrophils per unit volume of blood	Blood Cell Consortium	per nL	563,946
Eosinophil count	Count of eosinophils per unit volume of blood	Blood Cell Consortium	per nL	563,946
Basophil count	Count of basophils per unit volume of blood	Blood Cell Consortium	per nL	563,946
**Outcome data**				
All types of arrhythmia	Self-reported, aggregated occurrence of all types of arrhythmia	UK Biobank	logOR	462,933
Atrial fibrillation	Paroxysmal or permanent atrial fibrillation, or atrial flutter	Meta-analysis	logOR	1,030,836
Atrioventricular block	Includes ICD-10 codes: I440-I442 (First degree, second degree and third degree atrioventricular block), I443 (Other and unspecified atrioventricular block)	FinnGen	logOR	75,885
LBBB	Includes ICD-10 codes: I444-I445 (Left anterior and Left posterior fascicular block), I446 (Other and unspecified fascicular block), I447 (Unspecified LBBB)	FinnGen	logOR	75,392
RBBB	Includes ICD-10 codes: I450 (Right fascicular block), I451 (Other and unspecified RBBB)	FinnGen	logOR	75,369
Paroxysmal tachycardia	Includes ICD-10 codes: I47 (Re-entry ventricular, supraventricular, ventricular tachycardia and unspecified paroxysmal tachycardia)	FinnGen	logOR	57,999

Phenotype	Gender	Ancestry	IEU ID	Summary data URL	PMID	URL for detailed phenotype description
**Exposure data**						
Lymphocyte count	Males and Females	European	ieu-b-32	https://gwas.mrcieu.ac.uk/datasets/ieu-b-32/	PMID: 32888493	https://pubmed.ncbi.nlm.nih.gov/32888493/
Monocyte count	Males and Females	European	ieu-b-31	https://gwas.mrcieu.ac.uk/datasets/ieu-b-31/	PMID: 32888493	https://pubmed.ncbi.nlm.nih.gov/32888493/
Neutrophil count	Males and Females	European	ieu-b-34	https://gwas.mrcieu.ac.uk/datasets/ieu-b-34/	PMID: 32888493	https://pubmed.ncbi.nlm.nih.gov/32888493/
Eosinophil count	Males and Females	European	ieu-b-33	https://gwas.mrcieu.ac.uk/datasets/ieu-b-33/	PMID: 32888493	https://pubmed.ncbi.nlm.nih.gov/32888493/
Basophil count	Males and Females	European	ieu-b-29	https://gwas.mrcieu.ac.uk/datasets/ieu-b-29/	PMID: 32888493	https://pubmed.ncbi.nlm.nih.gov/32888493/
**Outcome data**						
All types of arrhythmia	Males and Females	European	ukb-b-3703	https://gwas.mrcieu.ac.uk/datasets/ukb-b-3703/	N/A	http://biobank.ndph.ox.ac.uk/showcase/field.cgi?id=20002
Atrial fibrillation	Males and Females	European	ebi-a-GCST006414	https://gwas.mrcieu.ac.uk/datasets/ebi-a-GCST006414/	PMID: 30061737	https://pubmed.ncbi.nlm.nih.gov/30061737/
Atrioventricular block	Males and Females	European	finn-a-I9_AVBLOCK	https://gwas.mrcieu.ac.uk/datasets/finn-a-I9_AVBLOCK/	PMID: 33318493	http://r2.finngen.fi/pheno/I9_AVBLOCK
LBBB	Males and Females	European	finn-a-I9_LBBB	https://gwas.mrcieu.ac.uk/datasets/finn-a-I9_LBBB/	PMID: 33318493	http://r2.finngen.fi/pheno/I9_LBBB
RBBB	Males and Females	European	finn-a-I9_RBBB	https://gwas.mrcieu.ac.uk/datasets/finn-a-I9_RBBB/	PMID: 33318493	http://r2.finngen.fi/pheno/I9_RBBB
Paroxysmal tachycardia	Males and Females	European	finn-a-I9_PAROXTAC	https://gwas.mrcieu.ac.uk/datasets/finn-a-I9_PAROXTAC/	PMID: 33318493	http://r2.finngen.fi/pheno/I9_PAROXTAC
