# Supplementary material for: Association between circulating leukocytes and arrhythmias: Mendelian randomization analysis in immuno-cardiac electrophysiology
Source: Front Immunol. 2023 Apr 5;14:1041591. doi: 10.3389/fimmu.2023.1041591 (PMC10113438; doi:10.3389/fimmu.2023.1041591)
Supplement: Supplementary file 2 [file DataSheet_2.pdf]

Table S2. Single-nucleotide polymorphisms (SNPs) used as instruments for differential leukocyte counts

Phenotype	Number of SNPs Identified in GWAS (P < 5 × 10–8)	Fraction of variance explained by SNPs	F-tatistic
Lymphocyte count	359	7.71%	131.098
Monocyte count	320	8.76%	169.097
Neutrophil count	299	5.91%	118.485
Eosinophil count	302	7.91%	160.253
Basophil count	137	2.57%	108.68
