# Supplementary material for: Association between circulating leukocytes and arrhythmias: Mendelian randomization analysis in immuno-cardiac electrophysiology
Source: Front Immunol. 2023 Apr 5;14:1041591. doi: 10.3389/fimmu.2023.1041591 (PMC10113438; doi:10.3389/fimmu.2023.1041591)
Supplement: Supplementary file 3 [file DataSheet_3.pdf]

Table S3. MR analyses evaluating the causal effects of leukocyte counts on arrhythmias after Steiger filtering

Exposure	Outcome	n	IVW		MR-Egger				Weighted median	
		SNPs	OR (95% CI)	p value	OR (95% CI)	p value	Intercept	Intercept p value	OR (95% CI)	p value
Neutrophil count	Atrial fibrillation	0	Unchanged							
	Atrioventricular block	0	Unchanged							
	Paroxysmal tachycardia	0	Unchanged							
	LBBB	0	Unchanged							
	RBBB	0	Unchanged							
Basophil count	Atrial fibrillation	0	Unchanged							
	Atrioventricular block	0	Unchanged							
	Paroxysmal tachycardia	0	Unchanged							
	LBBB	0	Unchanged							
	RBBB	0	Unchanged							
Eosinophil count	Atrial fibrillation	0	Unchanged							
	Atrioventricular block	0	Unchanged							
	Paroxysmal tachycardia	0	Unchanged							
	LBBB	0	Unchanged							
	RBBB	0	Unchanged							
Lymphocyte count	Atrial fibrillation	1	0.970(0.928-1.013)	0.174	0.973(0.887-1.067)	0.564	0	0.937	0.931(0.880-0.985)	0.013
	Atrioventricular block	0	Unchanged							
	Paroxysmal tachycardia	0	Unchanged							
	LBBB	0	Unchanged							
	RBBB	0	Unchanged							
Monocyte count	Atrial fibrillation	0	Unchanged							
	Atrioventricular block	0	Unchanged							
	Paroxysmal tachycardia	0	Unchanged							
	LBBB	0	Unchanged							
	RBBB	0	Unchanged							

n SNPs = number of outlier SNPs detected Steiger filtering and removed from the analysis. The term 'unchanged' refers to analysis results that remain the same before and after performing Steiger filtering.
