# Supplementary material for: Association between circulating leukocytes and arrhythmias: Mendelian randomization analysis in immuno-cardiac electrophysiology
Source: Front Immunol. 2023 Apr 5;14:1041591. doi: 10.3389/fimmu.2023.1041591 (PMC10113438; doi:10.3389/fimmu.2023.1041591)
Supplement: Supplementary file 4 [file DataSheet_4.pdf]

Table S4. Sensitivity analyses of causal relationships for combinations of exposures and outcomes whose causal estimates from IVW analysis were non-significant.

Exposure	Outcome	MR-Egger				Weighted median		MR-PRESSO			
		OR (95% CI)	p value	Intercept	Intercept p value	OR (95% CI)	p value	OR (95% CI)	p value	Global pleiotropy p value	Outliers deleted
Lymphocyte count	All types of arrhythmia	1.0001 (0.9985-1.0017)	0.889	1.23E-05	0.51	1.0003 (0.999-1.0015)	0.679	1.0006 (0.9998-1.0013)	0.132	0.418	0
Monocyte count	All types of arrhythmia	0.9992 (0.998-1.0004)	0.179	8.41E-06	0.608	0.9994 (0.9982-1.0006)	0.309	0.9994 (0.9988-1.0001)	0.079	0.935	0
Neutrophil count	All types of arrhythmia	0.9997 (0.9978-1.0016)	0.753	1.86E-06	0.93	0.9996 (0.9982-1.001)	0.579	0.9998 (0.9989-1.0006)	0.607	0.266	0
Eosinophil count	All types of arrhythmia	1.0011 (0.9995-1.0026)	0.194	-1.66E-05	0.428	1.0005 (0.9993-1.0018)	0.399	1.0005 (0.9997-1.0013)	0.217	0.357	0
Basophil count	All types of arrhythmia	1.0006 (0.9969-1.0043)	0.762	-2.82E-05	0.465	1.0001 (0.998-1.0022)	0.907	0.9993 (0.9979-1.0007)	0.312	0.422	0
Monocyte count	Atrial fibrillation	1.032 (0.972-1.095)	0.301	-0.001	0.877	1.025 (0.98-1.073)	0.278	1.028 (0.992-1.065)	0.129	<0.001	13
Eosinophil count	Atrial fibrillation	1.072 (0.988-1.164)	0.096	-0.002	0.057	1.011 (0.959-1.066)	0.686	1.001 (0.96-1.045)	0.946	<0.001	8
Lymphocyte count	Paroxysmal tachycardia	0.953 (0.644-1.411)	0.81	0.001	0.807	1.112 (0.81-1.526)	0.511	0.994 (0.819-1.207)	0.954	0.068	0
Monocyte count	Paroxysmal tachycardia	0.978 (0.743-1.287)	0.872	-0.002	0.715	1.035 (0.772-1.387)	0.819	0.939 (0.807-1.092)	0.413	0.979	0
Neutrophil count	Paroxysmal tachycardia	1.109 (0.705-1.744)	0.654	-0.003	0.561	1.124 (0.792-1.596)	0.512	0.985 (0.803-1.209)	0.885	0.765	0
Eosinophil count	Paroxysmal tachycardia	0.822 (0.564-1.197)	0.306	0.003	0.577	0.87 (0.643-1.177)	0.366	0.902 (0.748-1.087)	0.279	0.309	0
Basophil count	Paroxysmal tachycardia	1.162 (0.624-2.165)	0.635	-0.002	0.793	1.412 (0.881-2.264)	0.152	1.081 (0.819-1.427)	0.583	0.932	0
Monocyte count	Atrioventricular block	1.374 (0.899-2.1)	0.142	-0.005	0.414	1.116 (0.736-1.692)	0.606	1.193 (0.924-1.541)	0.178	0.132	0
Neutrophil count	Atrioventricular block	0.99 (0.483-2.028)	0.978	-0.004	0.667	0.754 (0.444-1.279)	0.295	0.861 (0.616-1.203)	0.381	0.068	0
Eosinophil count	Atrioventricular block	0.958 (0.549-1.674)	0.881	-0.003	0.726	0.946 (0.602-1.486)	0.809	0.879 (0.666-1.16)	0.363	0.315	0
Basophil count	Atrioventricular block	0.62 (0.242-1.586)	0.318	0.019	0.089	0.89 (0.434-1.826)	0.75	1.259 (0.789-2.007)	0.336	0.381	0
Lymphocyte count	LBBB	2.2 (0.853-5.673)	0.103	-0.015	0.209	1.26 (0.568-2.794)	0.57	1.298 (0.813-2.073)	0.276	0.161	0
Monocyte count	LBBB	0.631 (0.323-1.235)	0.179	0.021	0.038	0.803 (0.391-1.646)	0.549	1.113 (0.749-1.654)	0.597	0.682	0
Neutrophil count	LBBB	0.367 (0.12-1.12)	0.078	0.024	0.067	0.802 (0.349-1.841)	0.602	0.922 (0.546-1.555)	0.76	0.407	0
Eosinophil count	LBBB	1.407 (0.504-3.927)	0.514	-0.013	0.387	0.99 (0.467-2.102)	0.98	0.951 (0.57-1.585)	0.846	0.001	2
Basophil count	LBBB	0.65 (0.141-2.997)	0.581	0.009	0.638	0.939 (0.295-2.993)	0.915	0.894 (0.422-1.894)	0.771	0.539	0
Lymphocyte count	RBBB	1.694 (0.478-6.007)	0.414	-0.016	0.307	0.911 (0.321-2.586)	0.86	0.955 (0.515-1.773)	0.884	0.594	0
Monocyte count	RBBB	0.59 (0.231-1.512)	0.272	0.02	0.164	0.589 (0.217-1.598)	0.298	1.005 (0.569-1.776)	0.987	0.314	0
Eosinophil count	RBBB	1.067 (0.307-3.705)	0.919	0.015	0.387	2.649 (0.928-7.559)	0.069	1.718 (0.93-3.173)	0.085	0.583	0
Basophil count	RBBB	0.311 (0.033-2.901)	0.305	0.038	0.164	0.33 (0.066-1.644)	0.176	1.235 (0.409-3.731)	0.709	0.136	0
