# Supplementary material for: Association between circulating leukocytes and arrhythmias: Mendelian randomization analysis in immuno-cardiac electrophysiology
Source: Front Immunol. 2023 Apr 5;14:1041591. doi: 10.3389/fimmu.2023.1041591 (PMC10113438; doi:10.3389/fimmu.2023.1041591)
Supplement: Supplementary file 5 [file DataSheet_5.pdf]

Table S5. Reverse MR analyses evaluating the causal effects of arrhythmias on counts of leukocyte sub-populations

Exposure	Outcome	IVW		MR-Egger				Weighted median	
		OR (95% CI)	p value	OR (95% CI)	p value	Intercept	Intercept p value	OR (95% CI)	p value
Atrial fibrillation	Neutrophil count	1.008 (1.003-1.013)	0.001	0.993 (0.977-1.01)	0.444	0.001	0.305	0.997 (0.988-1.006)	0.529
	Basophil count	1.001 (0.996-1.006)	0.703	1.005 (0.999-1.011)	0.663	-0.002	0.388	1.001 (0.998-1.005)	0.484
	Eosinophil count	0.994 (0.989-1)	0.032	1.001 (0.996-1.006)	0.783	-0.005	0.105	0.999 (0.997-1.002)	0.498
	Lymphocyte count	0.995 (0.99-1)	0.04	1.007 (0.957-1.06)	0.078	-0.001	0.891	1.004 (0.991-1.017)	0.578
	Monocyte count	1.007 (1.002-1.011)	0.008	1.005 (0.999-1.01)	0.098	-0.002	0.132	1.003 (0.998-1.008)	0.261
Atrioventricular block	Neutrophil count	1.001 (0.998-1.004)	0.66	0.998 (0.974-1.022)	0.879	0.001	0.427	1.007 (0.996-1.017)	0.222
	Basophil count	1.002 (0.998-1.005)	0.348	1 (0.992-1.008)	0.08	0	0.93	1 (0.998-1.003)	0.675
	Eosinophil count	1 (0.997-1.003)	0.961	1.002 (1-1.004)	0.705	-0.004	0.006	1.001 (0.999-1.003)	0.526
	Lymphocyte count	1.001 (0.998-1.004)	0.436	0.992 (0.954-1.032)	0.478	0.003	0.553	0.997 (0.984-1.009)	0.606
	Monocyte count	1 (0.997-1.003)	0.873	1.003 (0.995-1.011)	0.927	-0.002	0.409	1.002 (0.998-1.007)	0.331
Paroxysmal tachycardia	Neutrophil count	1.006 (0.997-1.014)	0.195	0.988 (0.969-1.008)	0.232	0	0.458	1.001 (0.991-1.01)	0.917
	Basophil count	1.004 (0.995-1.013)	0.409	1.003 (0.997-1.008)	0.376	0.001	0.663	1.004 (1.001-1.008)	0.024
	Eosinophil count	1.001 (0.993-1.01)	0.787	1.001 (0.998-1.004)	0.469	-0.003	0.083	1 (0.998-1.003)	0.782
	Lymphocyte count	0.973 (0.965-0.981)	0	0.984 (0.941-1.028)	0.716	0.004	0.418	0.996 (0.984-1.008)	0.521
	Monocyte count	1.004 (0.996-1.012)	0.364	1.001 (0.996-1.006)	0.33	-0.001	0.611	1.002 (0.997-1.006)	0.444
LBBB	Neutrophil count	1.002 (1-1.005)	0.068	0.995 (0.972-1.019)	0.676	0	0.997	1.007 (0.997-1.016)	0.17
	Basophil count	1.003 (1-1.005)	0.038	1.001 (0.995-1.007)	0.707	-0.001	0.815	1.001 (0.998-1.004)	0.604
	Eosinophil count	1.004 (1.001-1.006)	0.004	1.001 (0.997-1.005)	0.716	-0.003	0.292	1 (0.998-1.002)	0.844
	Lymphocyte count	1 (0.998-1.003)	0.863	1.042 (0.835-1.301)	0.105	-0.016	0.525	0.999 (0.986-1.012)	0.886
	Monocyte count	1.001 (0.999-1.002)	0.38	1.004 (0.999-1.009)	0.791	-0.002	0.146	1.002 (0.997-1.006)	0.439
RBBB	Neutrophil count	1 (0.998-1.001)	0.704	0.999 (0.973-1.025)	0.913	0.001	0.384	1.001 (0.99-1.012)	0.898
	Basophil count	0.998 (0.996-1)	0.028	0.999 (0.989-1.008)	0.552	0.003	0.4	1 (0.996-1.004)	0.947
	Eosinophil count	0.999 (0.998-1.001)	0.517	1.001 (0.998-1.004)	0.667	-0.002	0.293	1 (0.998-1.002)	0.904
	Lymphocyte count	0.999 (0.997-1.001)	0.33	0.992 (0.957-1.028)	0.227	0.003	0.442	1.006 (0.994-1.018)	0.354
	Monocyte count	1 (0.998-1.001)	0.858	1.007 (0.996-1.018)	0.795	-0.004	0.165	1.003 (0.998-1.008)	0.231
